# Core decompression with autologous adult live-cultured osteoblast implantation for osteonecrosis of the femoral head: a prospective mid-term outcome analysis

**DOI:** 10.25122/jml-2024-0392

**Published:** 2025-07

**Authors:** Alok Chandra Agrawal, Ankit Kumar Garg, Shubham Bhardwaj, Harshal Sakale, Anupam Inamdar, Lohitesh Saparey, Rudra Narayan Dash

**Affiliations:** 1 Department of Orthopaedics, All India Institute of Medical Sciences, Raipur, Chhattisgarh, India

**Keywords:** osteonecrosis of the femoral head, stem cells, autologous adult live-cultured osteoblasts (AALCO)

## Abstract

Osteonecrosis of the femoral head (ONFH) is a challenging condition that mainly affects young and middle-aged adults, causing pain, disability, and joint collapse. Current treatment options include medications, physical therapy, and surgical interventions such as core decompression and total hip replacement. However, there is growing interest in regenerative medicine for managing ONFH. This study evaluated the outcomes of core decompression augmented with adult autologous live cultured osteoblasts (AALCO) in patients with early-stage ONFH. Patients diagnosed with ONFH, Ficat-Arlet Grades 1, 2, and 3, underwent a staged procedure involving bone marrow aspiration and the cultivation of 48 million osteoblastic lineage cells. Subsequently, this culture was injected following core decompression and curettage of the necrotic area in the femoral heads. Patients were then followed for 18 to 26 months and evaluated for radiological progression of the disease and changes in functional outcome using the Harris Hip Score (HHS) and Visual Analog Scale (VAS). Forty-eight hips (34 patients with 14 bilateral ONFH) were included in the study and followed up for 18 to 26 months. During this period, 29 patients (40 hips) exhibited progressive signs of healing, resulting in a significant improvement in the mean HHS and a reduction in VAS scores. Core decompression augmented with implantation of autologous live cultured osteoblasts is a reliable treatment approach for managing the early stages of ONFH in young patients caused by various factors. The method aims to halt disease progression through osteoblastic stem cell-mediated new bone formation, leading to improved functional outcomes and potentially delaying or avoiding the need for total hip arthroplasty.

## INTRODUCTION

Osteonecrosis of the femoral head (ONFH) is a serious condition that involves the death of bone tissue in the femoral head. This leads to pain, disability, and, eventually, joint collapse. The disease primarily affects young adults and can significantly diminish their quality of life. ONFH has various causes, including the use of corticosteroids, trauma, alcohol abuse, and certain medical conditions such as sickle cell disease. It predominantly impacts younger and middle-aged individuals, creating economic and social burdens by affecting the most productive age group [1-3].

Current treatment options for ONFH include medications, physical therapy, core decompression and autologous cultured osteoblasts, osteotomy, and total hip replacement in advanced stages [4-6]. As interest in regenerative medicine for treating ONFH grows, using autologous cultured osteoblasts has emerged as a promising new approach for managing this challenging condition [7].

One significant advantage of using autologous adult live-cultured osteoblasts for ONFH is their ability to promote long-term healing and regeneration of bone tissue. Unlike traditional treatments such as medication or surgery, which may only provide temporary relief, autologous cultured osteoblasts offer a more permanent solution by stimulating new bone growth in the affected area. This can help prevent further deterioration of the femoral head and delay or even avoid the need for joint replacement surgery.

Orthobiologics, such as platelet-rich plasma (PRP), mesenchymal stem cells (MSCs), and the application of other stem cell progenitors and biologics, have paved the way for better results through the implantation of osteoblasts in ONFH lesions. This study used autologous live-cultured osteoblasts concentrated in the ONFH of varied etiologies. We followed up with the patients to observe any changes in their functional outcomes. Osteoblasts were cultured from autologous bone marrow harvested from the patients and then implanted after core decompression. Patients were followed up for a minimum of 18 months to track improvements in functional outcomes and radiological progression of the disease.

## MATERIAL AND METHODS

Following approval from the Institutional Ethical Committee (AIIMSRPR/IEC/1919), a prospective study was conducted in accordance with the STROBE guidelines, between November 2020 and February 2023. Patients aged 18–60 years diagnosed with osteonecrosis of the femoral head, staged as Modified Ficat and Arlet grades I–III based on clinical evaluation, plain radiographs, and magnetic resonance imaging (MRI), were eligible for inclusion after providing written informed consent. They were further sub-grouped based on their etiological diagnosis. The patients with ONFH secondary to post-traumatic conditions were excluded from the study. The study strictly followed the rules per the 1964 Declaration of Helsinki and all its later amendments. A previous publication by the authors has reported a subset of this cohort with short-term (six-month) follow-up data [8].

## SAMPLE SIZE ESTIMATION

We conducted a power analysis based on a study by Changjun *et al*. [9] to estimate the sample size at 41, using an alpha error of 5% and a power of 95%.

### Surgical procedure

#### Preparation of autologous live cultured osteoblasts (AALCO)

All patients underwent a standardized two-stage surgical procedure. In the first stage, over 12 mL of bone marrow was aspirated from the posterior superior iliac crest and transported under cold chain conditions to Regrow Biosciences (Mumbai, India) for ex vivo expansion. Mesenchymal stem cells (MSCs) were cultured and differentiated into osteoblastic lineage cells through three passages. Forty-eight million autologous live cultured osteoblast concentrates were collected per case and transported back for implantation to maintain a strict cold chain. The quantity of the AALCO was determined based on a previous study [10] related to the osteocyte requirements for adult head size. OSSGROW (Regrow Biosciences Pvt Ltd., Mumbai, India) is a commercially approved Indian FDA technique that involves implanting autologous live cultured osteoblast concentrate, produced from bone marrow aspirate and mesenchymal stem cells, for the treatment of ONFH. The final product, containing a highly characterized homogenous cell population, was received from the laboratory after 3–4 weeks.

#### Core decompression and osteoblast implantation

**Figure 1:**
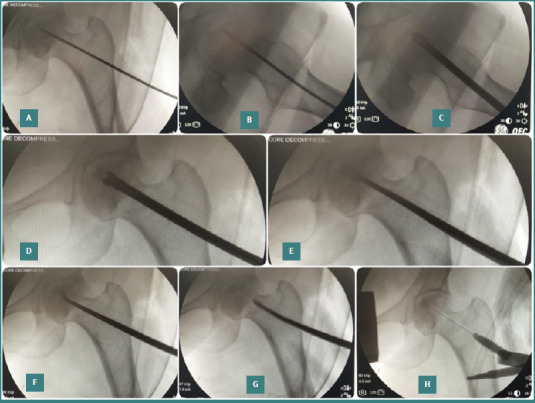
Intraoperative fluoroscopic images demonstrating the surgical technique. A–D, Core decompression using an 8-mm drill over a guide wire; E–G, curettage of sclerosed bone using curved curettes; H, delivery of AALCO using a TISSEEL kit through an 18G spinal needle and interference screw.

In the final operative step, under spinal anesthesia, patients were placed supine on a fracture table with the affected limb internally rotated by 15 degrees. The lesion was marked in both anteroposterior and lateral planes using 2 mm K wires under C-arm guidance. Core decompression was performed using an 8 mm cannulated drill over the guide wires, and the sclerotic bone at the lesion site was thoroughly removed using curettage. 4 A total of 4.8 × 10^6^ autologous live cultured osteoblasts was delivered into the decompressed area using a TISSEEL Kit (Baxter, USA) and an 18G spinal needle via an 8 mm interference screw. Finally, the tract was sealed with cancellous allograft obtained from a certified bone bank (Figure 1 A-G).

### Post-operative protocols

All patients were initially maintained on non-weight-bearing mobilization for 2 weeks, followed by partial weight-bearing for 1 week, and then progressed to full weight-bearing as tolerated. To optimize the biological healing response facilitated by AALCO, patients were advised to avoid non-steroidal anti-inflammatory drugs (NSAIDs), as these may interfere with the immune-mediated regenerative process.

### Outcome assessment

Demographic data, including age, gender, body mass index (BMI), and etiology of ONFH, were recorded for all patients. Staging was performed using the Modified Ficat and Arlet classification based on radiographic and MRI findings. Clinical and radiological evaluations were conducted preoperatively, with pain assessed using the Visual Analog Scale (VAS; 0–10, with 10 indicating the worst pain), and function assessed via the Harris Hip Score (HHS). VAS and HHS were recorded at baseline (preoperatively), 6 weeks, 6 months, 1 year, and the final follow-up. Annual MRI scans were performed to monitor structural changes in the femoral head, and any postoperative complications were documented throughout the follow-up period.

### Statistical analysis

The data were entered and analyzed using the Statistical Package for Social Sciences software, version 26.0 (SPSS Inc., Chicago, IL, USA). Continuous and categorical variables were expressed as means ± standard deviation (SD) and percentages. A paired *t*-test was conducted to compare the mean HHS score before and after the procedure. Two-sided *P* values were considered statistically significant at *P* < 0.05.

## RESULTS

Core decompression with implantation of autologous adult live-cultured osteoblasts was performed on 48 hips from 34 patients, of whom 14 had bilateral involvement. The mean age was 24.9 ± 5.4 years (range, 18–44 years). The cohort consisted of 22 men (65%) and 12 women (35%), with a mean follow-up duration of 22.4 ± 4.3 months (range, 18–26 months). Disease staging, based on the Modified Ficat and Arlet classification, ranged from stage I to III. Full demographic and baseline characteristics are provided in Table 1.

**Table 1:** Demographic data of participants

Variables	
Age	24.9 ± 5.4 years
Sex Male Female	22 (65%) 12 (35%)
BMI	25.7 ± 3.2
Joints involved Unilateral Bilateral	20 14
Staging Ficat Arlet Stage 1 Ficat Arlet Stage 2a Ficat Arlet Stage 2b Ficat Arlet stage 3	10 22 12 4
Etiology Idiopathic Sickle cell disease Sickle cell trait Steroids Alcohol consumption	3 6 12 9 4
Follow-up	22.4 ± 4.3 (range, 18–26 months)

A contrast was made between pre-operative and postoperative VAS pain scores, HHS at 6 weeks, 6 months, 1 year, and at final follow-up (Table 2). The pre-operative average score was 7.5, and the postoperative average score at 6 weeks follow-up was 2.5. At final follow-up, 94% of patients (*n* = 32) reported a greater than 50% reduction in pain.

**Table 2:** Comparison of pre-operative and post-operative HHS and VAS scores

	HHS score	VAS score
Pre-operative	48 ± 5.51	7.5 ± 2
Postoperative 6 weeks 6 months 1 year Final follow-up	80 ± 5.45 84 ± 5.32 86 ± 5.6 86 ± 3.2	2.5 ± 0.96 2.3 ± 0.7 2.1 ± 0.55 1.8 ± 0.33
*P* value	<0.001	<0.001

**Figure 2:**
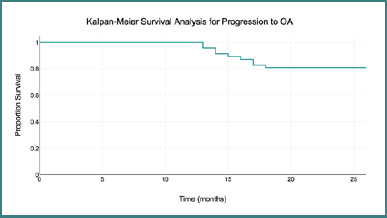
Kaplan-Meier survival analysis showing time to progression to osteoarthritis (OA) following core decompression with autologous adult live-cultured osteoblast implantation

**Figure 3:**
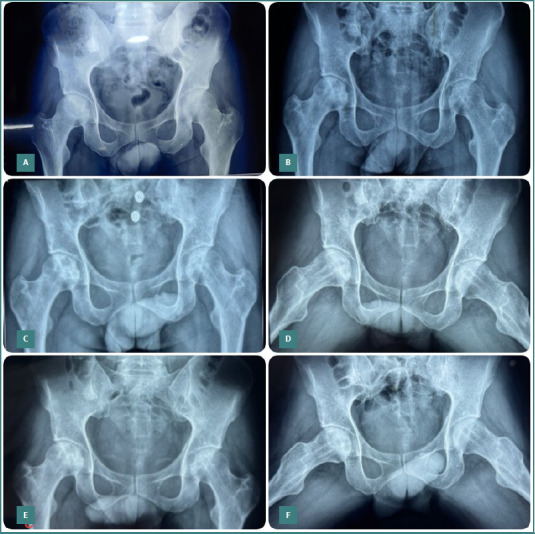
Serial anteroposterior pelvic radiographs showing bilateral hips of a patient with improved osteogenesis at the necrotic area and preservation of the spherical contour of the femoral head. A, Pre-operative radiograph; B, Six-month follow-up; C–D, Twelve-month follow-up; E–F, Twenty-four-month follow-up showing improved osteogenesis in the necrotic area and preservation of femoral head sphericity.

**Figure 4:**
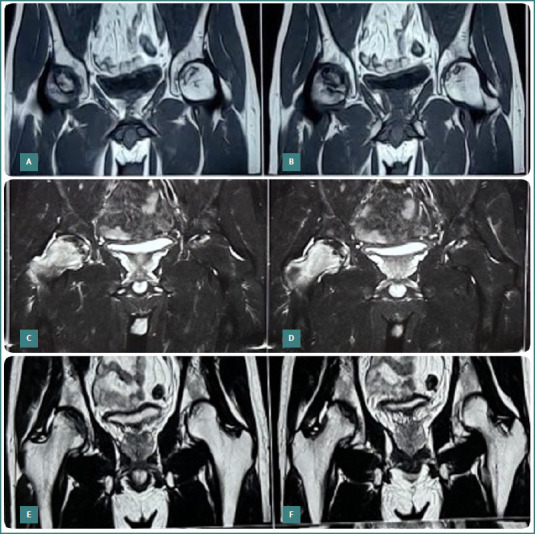
Serial MRI images of a patient treated with core decompression and autologous adult live-cultured osteoblast implantation. A–B, preoperative coronal MRI sections of the same patient with Ficat and Arlet stage IIb in the right hip and stage 2a in the left hip. C–F, MRI images at 24 months follow-up showing increased osteogenesis and signs of healing.

Postoperatively, 29 patients (40 hips) showed no overt progression in postoperative imaging after the decompression procedure, as observed up to the final follow-up (mean time, 22.4 months). Eight hips in five patients showed progression to osteoarthritis; four of these were initially classified as Ficat-Arlet stage IIb and four as stage III. These hips were scheduled for total hip arthroplasty (Figure 2). The radiological findings (both radiographs and MRI) showed improvement with significant osteogenesis and maintenance of the femoral head sphericity, as depicted in Figures 3 (A-F) and 4 (A-F). One patient had a surgical site infection, which was managed with debridement and STIMULAN application. No other infections or wound-healing complications were reported.

## DISCUSSION

ONFH is a gradually progressive disease that leads to severe disability among patients over time. Timely intervention during the pre-collapse stage of the femoral head, particularly through core decompression (CD), can prevent or slow the progression of disease. CD decreases intraosseous pressure, reduces edema, and improves circulation of the femoral head. Several procedural modifications have been explored, including single large-core drilling (8–10 mm) [11], multiple small-diameter drillings [12], and combinations with electrical stimulation [13], as well as various augmentation techniques such as vascularized and non-vascularized bone grafts, platelet-rich plasma, and bone marrow aspirate. These approaches yield variable success, with reported failure rates ranging from 20% to 70% [7,12,14-16]. The failure rates of CD alone have been attributed to a large core diameter, early postoperative weight-bearing, incomplete bone graft compaction, and deprivation of mesenchymal stem cells [17]. Song *et al*. [12], in their 5-year follow-up study, reported a survival rate of 79%, 77%, and 35% for Ficat stages I, II, and III, respectively.

Cumulative research over the last two decades has shown that a decreased number of osteoprogenitor cells in the bone marrow of the femoral head is closely associated with the etiology of ONFH, leading to interest in applying osteogenic precursor cells, such as autologous live-cultured osteoblasts, concentrated in the necrotic lesions of the femoral head. The recent decade of regenerative medicine has seen the use of mesenchymal stem cells to provide osteoprogenitor cell lineages, facilitating bone remodeling and repair in necrotic areas. Few studies have suggested that it also enhances the mobilization and homing capacity of mesenchymal stem cells [18,19]. The use of autologous live cultured osteoblast concentrate in combination with core decompression has been shown to improve clinical outcomes and slow down the radiographic progression of the disease.

Numerous research papers have been published on stem cell therapy for osteonecrosis; however, there are currently no standardized guidelines regarding the methods for harvesting, processing, and transplanting these cells. Various studies have utilized different types of stem cells, including bone marrow mesenchymal/mononuclear stem cells, peripheral blood stem cells, and human umbilical cord mesenchymal stem cells [18]. Hernigou *et al*. [7] first proposed using bone marrow mesenchymal stem cells in conjunction with core decompression. The relative ease of harvesting and processing these cells, along with the higher yield of various cell lineages, has contributed to their increasing use. Additionally, the number of cells collected in some studies has varied significantly, ranging from 90,000 to 3.46 × 10^9^ in volumes between 1 and 60 ml [18].

In our study, the mean age of the patients was 24.9 ± 5.4 years, with a male-to-female distribution of 22:12 and an average BMI of 25.7 ± 3.2, representing a relatively young and diverse patient population. All consecutive patients with ONFH, up to Ficat and Arlet stage IIB, were treated with core decompression augmented by autologous adult live-cultured osteoblast implantation. They showed marked improvement in their pain scores and HHS scores at each subsequent follow-up. The mean preoperative VAS pain score of 7.5 decreased to 2.5 postoperatively. Additionally, 29 patients (40 hips; 83.3%) showed no radiological progression at a mean follow-up of 22.4 months. However, five patients (eight hips), specifically four hips with Ficat and Arlet stage 2B, and all four hips with stage 3 disease, showed progression to osteoarthritis. Our study findings are consistent with previous studies [2,7,8,11,15,20-25].

A follow-up study conducted by Hernigou *et al*. [7] over a twenty-five-year period reported that stem cell therapy was more effective in reducing collapse and the need for total hip replacement compared to core decompression alone. Similarly, a ten-year follow-up study by Li *et al*. [26] reported improved subjective scores and a longer median survival time. A brief comparative outline is represented in Table 3 [6,8,25,27-30].

**Table 3:** Summary of clinical evidence on autologous adult live-cultured osteoblasts (AALCO)

Author	Year	Study design	Patients	Key findings	Conclusion
Kim *et al.* [27]	2008	Case report	2 hips (1 patient)	At 5-year follow-up, one femoral head showed remodeling; the contralateral side showed progressive degeneration.	AALCO demonstrated potential in halting disease progression in avascular necrosis AVN.
Palekar *et al.* [6]	2021	Case series	15 patients	Hip joints were preserved structurally by regaining the joint biomechanics after osteoblast implantation	Autologous osteoblast cell therapy is recommended in early AVN of the femoral head
Palekar *et al.* [28]	2021	Retrospective study	101 hips (64 patients)	Total hip arthroplasty (THA) was delayed in 71.3% of cases; 71.1% of early-stage (I & II) patients improved versus 58% in late-stage (III & IV).	AALCO preserved the natural hip joint and reduced progression, especially in early stages.
Sadat-Ali *et al.* [29]	2022	Prospective study	63 patients	MRI at 2 years showed new bone formation and reduction of avascular lesions	AALCO showed strong potential for AVN healing.
Agarwal *et al.* [8]	2023	Case series	6 patients	There was a significant improvement in the quality of life and daily activities after the implantation of AALCO. The necrotic area of the femoral head did not increase in size.	AALCO implantation resulted in reduced pain and improved hip function in cases of AVN associated with sickle cell anemia.
Shankar *et al.* [30]	2023	Case report	1 patient	At 6 years, the AALCO-treated hip remained viable with maintained sphericity, while the contralateral BMAC-treated hip showed deterioration.	AALCO is an effective biological option for treating avascular necrosis of the femoral head.
Patro *et al.* [25]	2024	Prospective study	41 hips (26 patients)	MRI at 3 years showed osteogenesis in 22 patients; 4 cases progressed to Grade IV and required THA.	AALCO with core decompression is effective for early-stage AVN.
Present study	2024	Prospective study	48 hips (34 patients)	At mean follow-up of 22.4 months, 29 patients (40 hips) showed signs of healing.	The early treatment of ONFH through core decompression using AALCO promotes physiological bone remodelling and slows disease progression.

The safety of autologous cultured osteoblast implantation is well established. Only one of our patients developed a wound infection, which was successfully treated with debridement. There were no reports of any allergies or reactions associated with the study. Similarly, Yan *et al*. [16] also reported a similar site infection.

The primary limitation of this study was the absence of a control group, which restricted the ability to directly compare outcomes and assess the natural progression of ONFH. Future research with larger sample sizes and extended follow-up periods is necessary to confirm these findings and establish this technique as a potential treatment option for ONFH. A key strength of this study lies in its uniform treatment approach, as all patients underwent core decompression augmented with autologous cultured osteoblast concentrate. The protocol was highly standardized, including the preparation of 4.8 × 10^7^ viable osteoblasts per patient, strict cold-chain maintenance, and consistent surgical delivery of 4 cc of the cell concentrate into the necrotic lesion of each femoral head under intraoperative imaging guidance.

## CONCLUSION

Early intervention in osteonecrosis of the femoral head using core decompression combined with biological therapies such as autologous cultured osteoblast implantation may promote physiological bone remodeling and delay disease progression. Future controlled studies with larger sample sizes are warranted to establish the role of ortho-biologicals in halting the natural progression of osteonecrosis of the femoral head.

## Data Availability

Further data is available from the corresponding author upon reasonable request
